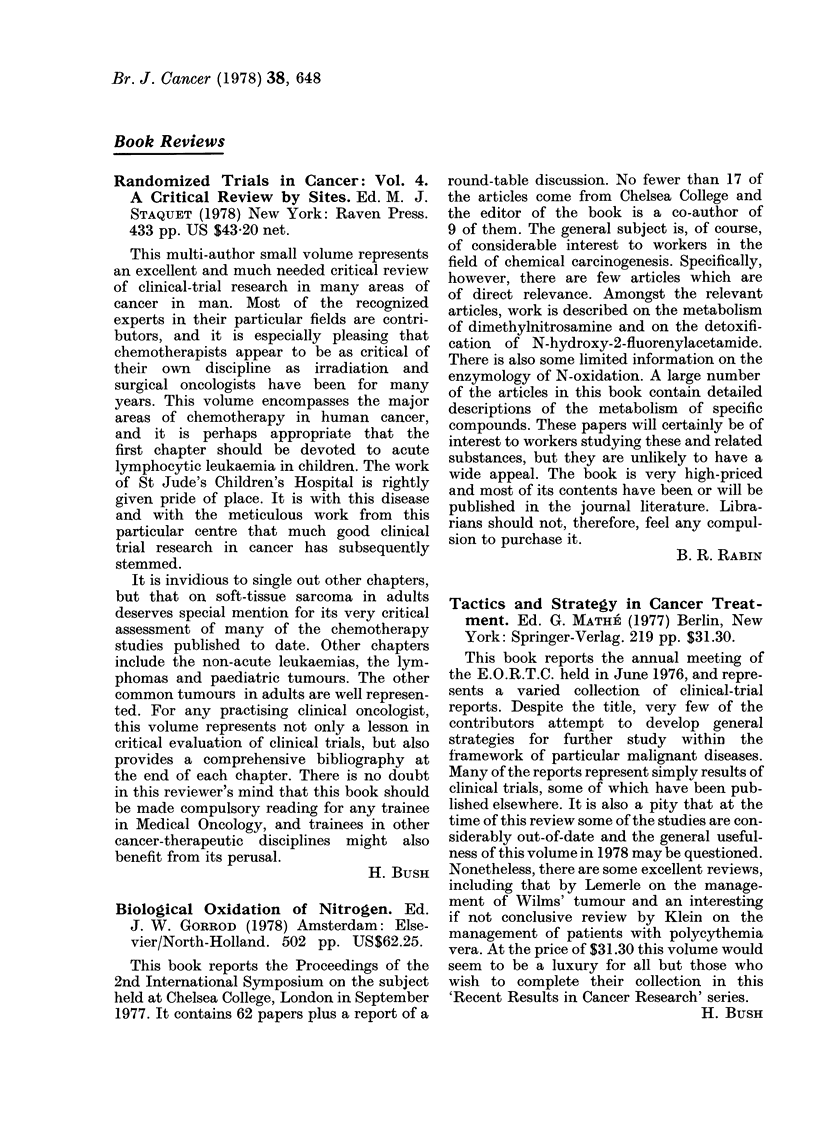# Randomized Trials in Cancer: Vol. 4. A Critical Review by Sites

**Published:** 1978-11

**Authors:** H. Bush


### *Book Reviews*

**Randomized Trials in Cancer: Vol. 4. A Critical Review by Sites.** Ed. M. J. Staquet (1978) New York: Raven Press. 433 pp. US $43·20 net.

This multi-author small volume represents an excellent and much needed critical review of clinical-trial research in many areas of cancer in man. Most of the recognized experts in their particular fields are contributors, and it is especially pleasing that chemotherapists appear to be as critical of their own discipline as irradiation and surgical oncologists have been for many years. This volume encompasses the major areas of chemotherapy in human cancer, and it is perhaps appropriate that the first chapter should be devoted to acute lymphocytic leukaemia in children. The work of St Jude's Children's Hospital is rightly given pride of place. It is with this disease and with the meticulous work from this particular centre that much good clinical trial research in cancer has subsequently stemmed.

It is invidious to single out other chapters, but that on soft-tissue sarcoma in adults deserves special mention for its very critical assessment of many of the chemotherapy studies published to date. Other chapters include the non-acute leukaemias, the lymphomas and paediatric tumours. The other common tumours in adults are well represented. For any practising clinical oncologist, this volume represents not only a lesson in critical evaluation of clinical trials, but also provides a comprehensive bibliography at the end of each chapter. There is no doubt in this reviewer's mind that this book should be made compulsory reading for any trainee in Medical Oncology, and trainees in other cancer-therapeutic disciplines might also benefit from its perusal.

H. Bush